# Ethnic minority patients’ experiences of filling in translated patient-reported outcome measures

**DOI:** 10.1177/22799036251390945

**Published:** 2025-12-18

**Authors:** Lise-Merete Alpers, Ingrid Hanssen

**Affiliations:** 1 Faculty of Health Sciences, VID Specialized University, Oslo, Norway; 2 Lovisenberg Diaconal University College, Oslo, Norway

**Keywords:** migrants, PROMs, chronic pain, culture, language

## Abstract

**Background:**

Problems assessing migrant patients’ pain may cause inadequate treatment. Standardised patient-reported outcome measures (PROMs) are used as basis for tailoring treatment plans. This study investigates patients’ experiences of filling in PROMs translated into their home language.

**Design and methods:**

Explorative design with in-depth interviews with 12 patients speaking the target languages Urdu, Somali, Arabic and Polish. The data analysis was inductive, thematic and hermeneutic in character.

**Results:**

Many interviewees found concepts used in the PROMs difficult to understand. Several viewed questions about mental health, suicide and sexual activity as taboo. Some of the PROM questions/answer options did not fit their situation and/or experience. Completing six different PROMs was exhausting.

**Discussion:**

Idiomatic and dialectic variations as well as diverse cultural backgrounds influence how concepts and questions were understood and made completing the PROMs challenging. Translations, although technically correct, must be understandable in the cultural context in which they are to be used. Some questions/response options do not reflect lived experience, and/or are couched in language reflecting biomedical understanding of illness. Culture, religion, traumatic experiences, migration-induced stress etc. shape individuals’ understanding and expression of health problems and symptoms. Secrecy and not sharing information is often an adaptive response to avoid shame.

**Conclusion:**

Although the PROMs used were translated by professional translators, cultural, religious and educational backgrounds influence how concepts and questions are comprehended. Questions considered too private/taboo might be ignored. These problems may lead to seriously impaired treatment outcomes.

## Introduction

Pain is part of the human experience, a fact that makes us “tend to assume that communicating about pain will go seamlessly across cultural boundaries.”^1^ In recent decades, however, researchers have come to understand that pain is a much more diverse concept than previously recognised, encompassing not only the notion of pain as an individual experience,^2,3^ but also highlighting the “racial and ethnic differences in the experience of pain.”^4^ Moreover, as chronic pain is more prevalent, widespread, and severe among migrants compared to others,^5^ and it is more challenging for healthcare professionals to assess the pain levels in patients who come from a different cultural and linguistic background than their own,^6,7^ this increases the risk of inadequate treatment for migrants’ pain.^8^

## Background

This study was conducted at a Norwegian University Hospital’s outpatient clinic for patients with severe chronic pain. Chronic pain (i.e. pain lasting 3 months or longer) may affect patients’ mood, thinking and behaviour. It is often linked to activity limitations, anxiety, depression, insomnia, fatigue and reduced quality of life.^9^

The clinic’s patients are asked to fill in several standardised questionnaires, so-called patient-reported outcome measures (PROMs), before seeing the treatment staff. PROMs provide status reports of patients’ “health condition that comes directly from the patient, without interpretation of the patient’s response by a clinician or anyone else.”^10^ The completed PROMs are “used to objectively assess a patient’s perception of pain across multiple dimensions”^11^ and as basis for the development of tailored treatment plans.

The pain clinic’s staff found that many migrant patients had problems coping with the various PROMs. This generated the need to investigate (1) challenges concerning the translation of PROMs according to the “gold standard” of forward-and-back translation^12^ and (2) whether the translations were linguistically and culturally congruent and thus, readily understandable for the clinic’s patients. The PROMs translated for this study were:

- Modified Oswestry Disability Index
- Location of the patient’s pain on body sketch
- Pain characteristics
- EQ-5D-5L
- Hopkins symptom checklist-25 (HSCL-25)
- Bodily Distress Syndrome

The content of these six PROMs will not be discussed. The four main languages spoken by the migrant patients at the clinic – Urdu, Somali, Arabic and Polish – are this study’s target languages.

The primary research question is: How may migrant pain clinic patients experience filling in PROMs translated into their home language? This led to a second question: What does the interviews tell us about the linguistic and cultural congruency, and thus, the validity of the translated PROMs?

## Method

We have previously^12^ described how the PROMs were translated and problems found during this process (Table 1).

**Table 1.** Translation steps and validation.^12^

Step 1	Step 2	Step 3	Step 4	Step 5	Step 6
Literature searches regarding translation methodology	Forward translations by professional translators (Norwegian →target languages)	Back translations by other translators (target languages →Norwegian)	Norwegian back-translations were reviewed by the first author and a physician at the pain clinic	Differences between the original and translated versions were discussed with translators to ensure maintenance of meaning	Interviews with three independent professional translators on general challenges regarding translations
	Discussions with the professional translators during work about linguistic challenges				

In this second part of the project, an explorative design was used to investigate whether migrant patients experienced the PROMs intelligible and straightforward to fill in. A main purpose was to validate the translations’ linguistic and cultural congruency. This was done by going through the translated PROMs with native speakers of the respective target languages and discuss possible challenges of comprehensibility.

### The interviews

The interviews were conducted in Norwegian by the first author. They took place in a quiet room at the pain clinic after the interviewees had completed the questionnaires and before their consultation with healthcare professionals. The completed PROMs served as an interview “guide.” The interviewer went through every question with each of the patients to learn how they understood the questions, and when needed, help find a response which they found to be more appropriate to their situation. A professional interpreter assisted at four interviews. For one of the patients who was illiterate, the interpreter read the questions and answer options. The digitally recorded interviews lasted 27–69 min. After 12 interviews, we considered data saturation to have been reached, as no new themes emerged.^13^

### Data analysis

The audio recorded interviews were transcribed verbatim by the first author and resulted in 118 single-spaced pages of data. The analysis was based on the Norwegian text and was inductive, thematic and hermeneutic in character, conducted collaboratively and manually, following the methodological steps of Braun and Clarke’s^14,15^ thematic analysis.

As pointed out by Braun and Clarke^14,15^ and Olmos-Vega et al.,^16^ reflexivity is essential in qualitative data analysis. Researchers must constantly be vigilant against the limitations imposed by imperceptible habits of thought.^17^ It is also important to reflect on personal experiences and previous research. The authors are both female nurses with PhDs and experienced qualitative researchers with intercultural research backgrounds. This background may have influenced our expectations regarding the findings. However, we will hold that possible researcher bias is reduced by the interviewer’s questions being guided by the clinic’s PROMs.

The goal of thematic analysis is to develop patterns or themes in the interview texts through six phases^14^:

*Phase 1*: We *familiarised* ourselves with the data, independently reading the data several times. This created a circular investigation of the texts as each reading led to deeper understanding of semantic nuances and overt meanings. We documented our individual thoughts and impressions, all the time checking that these reflected what was said in the interviews.
*Phase 2: Data coding*. We reviewed the transcribed texts collaboratively, which facilitated the extraction of initial codes from the dataset.
*Phase 3: Generating initial themes.* We identified shared patterns of meaning across the dataset. This facilitated compiling clusters of codes that shared core ideas or concepts. Similar features and relationships were determined, and their relevance to the research question was thoroughly discussed.^14^
*Phase 4: Developing and reviewing themes* involved assessing the candidate themes against the coded data that were relevant to each theme, thus determining whether they accurately represented the meanings apparent in the entire dataset.
*Phase 5: Refining, defining, and naming themes.* We decided on what headings would reflect the various paragraphs/themes in the finished paper text, fine-tuning the analysis to ensure that each theme was distinctly delineated, “built around a robust core concept or essence.”^15^
*Phase 6*: We completed the analytic process by *writing up this paper*, where the acquired empirical data and the ensuing analysis were explained, clarified and discussed.

**Figure 1.**
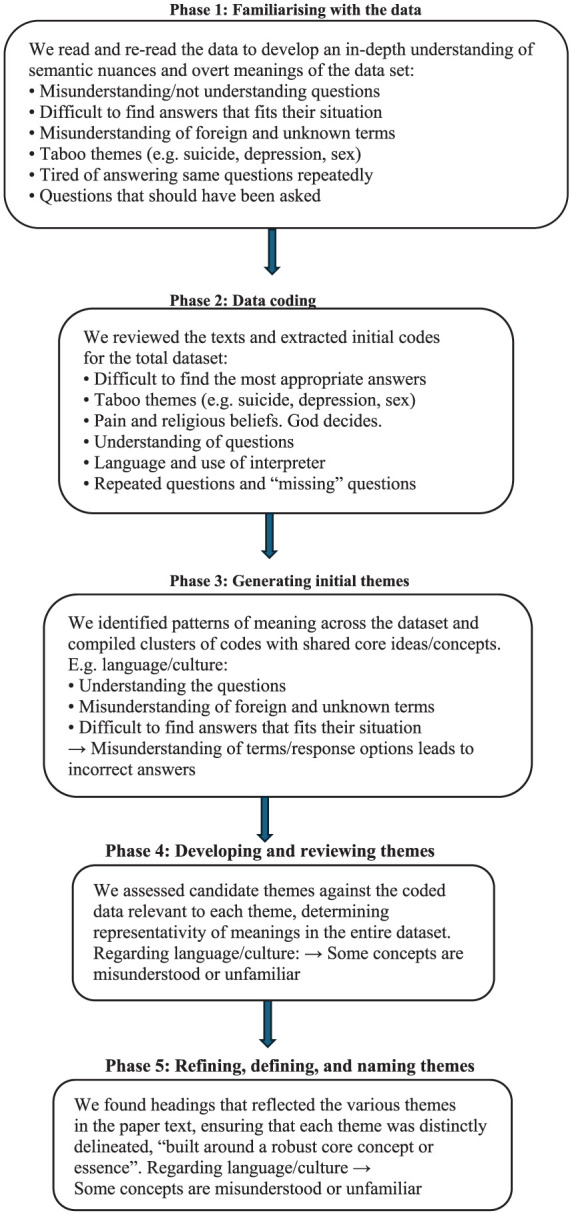
Illustration of the analysis process.

An illustration of the analysis process is presented in Figure 1.

### Rigour, credibility, trustworthiness and transferability

Rigour was achieved by following Braun et al.’s^18^ analytic phases. Being two analysts enhanced the study’s credibility and rigour, as did presenting direct quotations to demonstrate that our analysis was grounded in the interviewees’ own descriptions of their thoughts and experiences.^13^ This also established trustworthiness and helped avoid bias. Thick descriptions provide readers with insight into how our findings may be applied to similar situations and contexts. The quotations presented in the result section were translated into English from Norwegian.

### Ethical considerations

Approval was obtained from the hospital’s Privacy Ombudsman for Research (case No. 18/03967). After the interviewees were informed orally and in writing that participation was voluntary and that they were free to withdraw at any time,^19^ informed consent was obtained. Confidentiality was maintained throughout the research process. The audio recordings were deleted following transcriptions, and the transcriptions were stored according to Norwegian research ethics guidelines.^19^ The interviews were planned so that the patients had time to rest before their consultation with the healthcare team. Throughout the interviews the interviewer checked how the patients were feeling as several of them expressed that their pain made answering the questions tiring.

## Results

Purposive sampling was used to recruit “information-rich” interviewees who possessed pertinent experiences related to the phenomenon studied.^20^ Potential interviewees were selected by the clinic’s healthcare staff during weekly meetings when they reviewed referral requests. Inclusion criteria: (1) first-generation migrants whose mother tongue was Polish, Somali, Urdu or Arabic; (2) at least 18 years of age; and (3) were able to complete an interview. Proficiency in Norwegian was not required, as interpreters were called in when needed. Exclusion criteria: patients with other home languages, cognitive or speech impairments and/or were under 18 years of age.

Based on these criteria, 36 patients were invited to participate in the study by letters. Although we wanted variations in linguistic backgrounds, initially only one Urdu speaker chose to participate. However, the clinic staff helped us recruit two more who found it interesting to join. Thus, we had 12 interviewees – eight men and four women – three representing each of the target languages. These had lived in Norway 4–46 years and were between 30 and 73 years of age (mean age: 48.5) (Table 2). The educational level varied from no formal schooling (illiterate) to completed upper secondary school, and one had a university degree.

**Table 2.** The interviewees.

Languages	No. of interviewees		Interviewee no.	Average age	Time in Norway	Needed interpreter
	Women	Men				
Polish		3	4–6	47.6 years	15–20 years	1
Somali	2	1	10–12	50.6 years	9–29 years	2
Urdu	2	1	7–9	55.0 years	30–46 years	1
Arabic		3	1–3	40.6 years	4–29 years	

During the data analysis, four main themes were identified: (1) Misunderstood or unfamiliar concepts; (2) Questions and/or response options do not fit the interviewees’ situation; (3) Taboo topics; (4) Answering several PROMs was exhausting, annoying, or overwhelming.

Initially, most of the interviewees stated that responding to the translated PROMs was unproblematic. However, when going through the various PROMs, it emerged that several had either avoided questions they had not understood, had misunderstood question, or were uncertain whether they had selected the response option that best described their situation. The talk during the interview helped them understand the meaning of the questions and/or determine which response was most fitting for them.

### Misunderstood or unfamiliar concepts

Several of the concepts used in the PROMs were confounding and often misunderstood. For instance, a patient (10) who had lived 9 years in Norway thought that “kilometres” (km) referred to time when answering how far she could walk. *Hyperventilation* was a concept misunderstood by several, as they associated it with a physical reaction only. An Urdu-speaking man with more than 20 years in Norway interpreted the concept as “breathing fast, like when one is tired. Like the way you breathe when running very fast.. . . Sometimes I get like that” (12). Others said that “I think that it is to have problems breathing, like not being able to breathe properly. Someone with sick lungs” (11) or “when one walks long distances, one becomes a little out of breath. Of course, I understand that it has something to do with the physical condition, not the psychological” (5). A Pakistani woman answered that she was hyperventilating because “when I leave the room after prayer, as soon as I come out, my throat gets very dry and my lips become very dry, too” (9).

For some interviewees questions concerning anxiety were challenging. For instance, a patient (9) wondered whether anxiety and depression are the same thing, and a Somali interviewee expressed that both good and bad are in God’s hands. She explained that for those who accept their fate and believe in God and live close to Him, questions concerning one’s mental state are incomprehensible (10).

### Questions and/or response options do not fit the interviewees’ situation

Some of the PROM questions or answer options did not fit interviewees’ situation and/or experiences. In the introduction to one of them, for instance, patients are asked to check the box which describes their condition best “as it is today.” Because pain intensity and experience of health may vary quite a lot during the day and from day to day, several found this question difficult to answer, as “today” was perceived as referring to a rather permanent state.

Finding satisfactory response options became even more difficult when questions on variations in pain intensity were coupled with other health problems. An example is the interviewee who suffered from fibromyalgia, joint pains and headaches that varied in strength. She found it difficult to indicate the pain intensity: “I can say [answer option] No. 4, but it is not like that all the time. It kind of comes in waves” (2). This patient also suffered from sensitivity to light, which was not included in the questionnaires. Others, too, found that many of the questions did not cover their situations. A patient with complex regional pain syndrome (CRPS) in an arm said regarding a question on ability to do heavy lifting: “I wonder whether it applies to lifting with my left arm or lifting things in general . . . because I have no problem lifting with my right arm” (4).

Others found some response options imprecise as symptoms and problems could be caused by side effects of medications and not solely by the diagnosis. For example, questions in two of the PROMs touched upon whether the condition affected their sexual activities. A Polish interviewee found these questions unclear because “the pain itself does not affect my sexual activity, but the [pain] medication does” (8). A couple of PROMs include questions about the extent to which patients experience low energy and/or concentration difficulties. Here, also, patients were uncertain whether they should respond affirmatively if these issues were due to side effects. One explained that “When it comes to tiredness, that I do experience. When it comes to concentration, I take Tramadol [an opioid], which makes it so I cannot concentrate” (5).

Some expressed frustration at having to waste time and effort answering questions they perceived as unrelated to their condition or for which no suitable reply option was provided. As one put it: “It is not necessary to ask a particular question if one doesn’t have an illness that concerns this theme, or one hasn’t complained about the parts of the body concerned . . . When I have told you that I have intense back pain and that I cannot walk because of this pain, you ask me, ‘Are you depressed? Do you experience anxiety?’ I wonder why you ask these kinds of questions” (10). The same interviewee indignantly pointed out that “I have pain and that kind of thing, and then you ask me about something totally different, whether I am worried.. . .That is something totally different.”

### Taboo topics

Questions regarding mental health, suicide and sexual activity had been left unanswered by several interviewees as they judged them to be inappropriate or taboo. Among those who did answer them, their views varied. While some found it natural to talk about mental health problems, others held that “one might become afraid by being asked about one’s mental state if one does not have any psychological problems. Some questions concerning anxiety and depression are difficult to answer because they are personal” (11). Others saw it as a positive thing to be asked about their mental health. An Arabic speaker (7) stated that when one is ill, worried, and depressed, one may feel better when talking about one’s problems. Despite agreeing with this, some worried that physicians and nurses might not observe their professional confidentiality, while others expressed their trust in the healthcare professionals.

While suicide was a topic some patients found to be taboo, others found it unproblematic and even helpful to talk about. Some Muslim patients saw suicide in light of religion. Particularly when a person has “a connection with God. . .. We die at the time decided by God” (10), this was seen as an unseemly topic to bring up. Others held that “it is a good question to ask” (6). A Pakistani interviewee (2) found it important to talk about her suicidal thoughts with healthcare professionals but not with family members. Several agreed with this, because if someone indicates that they have such thoughts, they can receive help “before they do something” (5). According to a Somali woman, however, if people struggle with such thoughts, they may have problems answering questions about suicide and one should check their mental state and assess whether to broach the topic or not (10).

A male interviewee (1) held that gender was important, especially when it came to interpreters, as it would be difficult for him to discuss suicidal thoughts with a female interpreter present. Having such conversations with a male interpreter would be ok. Also, other patients found questions about one’s sexual activities difficult to discuss with healthcare professionals of the opposite sex. Particularly highly religious Muslim men may shy away from being questioned about sexual problems by young women: “Some may perceive this as a very intimate question, and they will perhaps decline to answer” (4). An elderly married woman said, “It is something I keep to myself; it is sensitive. It is something one doesn’t want to talk about” (9). A widowed patient held that one could ask such questions “if you have a partner, a husband or wife . . . but it is wrong to ask people like me, who do not have a partner” (10). This latter sentiment was echoed by several interviewees. Some said that they may discuss such things with healthcare professionals, while others stated that they could talk to certain family members, such as their spouse, but no one else. Others claimed that “it is quite normal. It is a part of life. It is no problem” (11). One added that this is “a good question, for in a way it is about pain. I therefore answered this question quite thoroughly” (4).

### Answering several PROMs was exhausting, annoying, or overwhelming

To be expected to complete six different PROMs, each with many questions, may be experienced as exhausting by any patient. For individuals with minimal education or scant reading skills, this task may be utterly overwhelming. “There are so many questions here. So many words” (3). Moreover, answering questions that were more or less identical in the various PROMs was both tiring and annoying: “Am I supposed to answer this again?” was a common reaction. Some suggested that many questions should have been merged to save time and effort. It was also frustrating to have to fill in the same PROMs every time they visited the clinic: “The same questions again and again . . . I have had back problems for three years and the same questions every time” (6).

## Discussion

This study demonstrates that many migrants may find completing PROMs challenging, regardless of background, as many questions are lacking in linguistic and cultural congruency. Based on our results, the following themes are discussed: a) need for clarification of unfamiliar or misunderstood concepts; b) when questions or response options make responding to PROMs difficult; c) taboo topics; and d) answering several PROMs may be exhausting, annoying, or overwhelming.

### Need for clarifying unfamiliar or misunderstood concepts

Differences in expression, linguistic nuances and cultural backgrounds may make it challenging for migrant patients to comprehend PROM questions even when correctly translated. Great idiomatic variations between languages, as well as diverse cultural backgrounds and dialectic expressions among speakers of a given language, influence how concepts and questions are understood.^21^ Hence, a lexically correct translated text is not necessarily linguistically and culturally congruent.^12^ Patel et al.^11^ found in their comparison between English-speaking and Arabic-speaking pain patient cohorts that cultural influences affected how participants appraised their pain experiences and how they responded to the questionnaires administered in their respective languages.

If concepts are not “in common lay language,” the PROMs will have “suboptimal meaning or readability for the majority of the general population.”^22^ In our 2022 study we found that the translators struggled to find solutions when an exact translation of a concept did not exist in the target language. In Somali, for instance, the concept “hyperventilation” created a problem. It was first translated as “breath” by the professional translator, but to promote understanding, “unusually quickly/often” was added in parentheses. Another concept that lacked a similar term in some languages was *anxiety*.^12^ An interviewee asked what anxiety was and whether it was the same as depression.

These were not only linguistic problems. Translations, although technically correct, must also be understandable in the cultural context in which they are to be used. One may for instance see a health problem like hypertension as caused by fate,^23^ as illustrated by the patient who gave religion as a reason for not understanding certain questions. For some, this may lead to religious healing measures like prayer instead of seeking professional medical advice. As to this, Jamil et al.^24^ maintain that education is needed to help patients who hold fatalistic beliefs adhere to their medical regimens.

Although Jamil et al.^24^ point to the importance of health education, our study shows that if the problem is the translated text’s linguistic and/or cultural inadequacy, the inability to understand the questions should not be attributed to the patients. Furthermore, although Nikolovski et al.^25^ point out that culturally compatible translations of questionnaires, such as PROMs, are invaluable for reflecting patients’ cultural understanding of health and illness and thus, crucial for enhancing decision-making in healthcare, Patel et al.^11^ emphasise the need for caution when utilising different language versions of a given PROM, as the same tool may yield different meanings across cultures.

### When questions or response options make responding to PROMs difficult

Other reasons why some PROM questions or response options might be difficult to answer besides problems of language and culture, are (1) The specificity of the questions do not reflect lived experience and/or (2) the PROMs reflect biomedical understanding of illness only.

Regarding the first issue, to answer how they feel “today” proved to be difficult as chronic pain tends to vary in strength and impact throughout the day. This creates the problem of which experience of today’s pain is “the correct” answer. As seen in the result section, one interviewee felt that he easily could have checked off in four different boxes. Furthermore, several interviewees complained that some response options did not reflect their experiences at all or did not addressed issues they would have liked to call attention to.

Regarding the second issue, culture and religion may shape both how health problems are understood and how symptoms are expressed. Religion may influence how pain is experienced, interpreted, and coped with.^26^ This is reflected in our findings through patients who experience pain as part of their spiritual life. For some patients, this may make it as important to focus on their religious faith as on other needs. Koffman et al.^27^ hold that Afro-Caribbeans, for instance, often perceive illness and pain as trials or tests of faith. Thus, health problems may confirm and strengthen the sufferers’ religious beliefs and loyalty to God. Research indicates that health professionals who ignore or undervalue such religious and/or cultural perspectives may compromise the management of the patient’s pain.^28^ Therefore, it is essential to understand what significance pain may have for patients socially, psychologically, culturally, and spiritually.^29^

Also, traumatic experiences earlier in life, migration-induced stress, longing for their home country, traditional gender roles, and high expectations to function normally despite chronic pain may all be factors which exacerbate pain.^30^ A case-control study examining 200 Iranian patients with back pain showed that education influenced how the patients reacted to chronic pain, as the patients with little education tended to have more accompanying psychosocial symptoms.^31^ As an explanation the authors suggest that those with higher education better understood the origin of their pain and had a larger repertoire for coping. As one of our interviewees put it, “There are some who do not manage so well, you know, who are not able to read so much.”

### Taboo topics

Questions concerning mental health, suicide and sexuality may be perceived as private and/or taboo and hence, difficult to answer. How their pain affects sexual activities was for instance a type of question that some found inappropriate to ask persons who were unmarried or without a partner. The interviewees most likely also needed to consider whether they could trust the health carers to honour their professional confidentiality, which in many cultures is not self-evident. Some seemed to worry that nurses and other healthcare professionals might not comply with this ethical and legal duty, while others expressed that they trusted them.

Different explanatory models, a lack of understanding of mental illness and stigma and taboos are recognised as barriers to seeking treatment for mental illness among migrants.^32^ Many migrant patients may discuss mental problems with close family members, but may be hesitant to seek help outside of the family in fear of damaging the family’s reputation.^33^ In collectivistic societies the family’s reputation, honour and social respect tends to be paramount.^34^ Secrecy and refraining from sharing information seen as shameful is often an adaptive response to avoiding shame.^35^ This may quell any inclination to discuss topics judged to be taboo with healthcare professionals.

Also, religion may influence the willingness to discuss private topics. For some patients, it may be equally important to focus on their religious faith as on their physical needs. Chan et al.^36^ claim that “other ways of knowing and understanding [the] experiences of illness” may be invalidated if spiritual and psychosocial factors are not acknowledged. Furthermore, the Quran forbids physical contact between men and women who are unrelated or not in familial relationships. For some, this may make it particularly challenging to discuss private matters with healthcare professionals of the opposite sex. In an Indian study that used the Oswestry Disability Index they found that almost 60% of the patients did not respond to questions related to sexual activity.^37^

Explaining beforehand that some questions among the PROMs they are asked to fill in may appear overly private may perhaps help patients address such issues. Discussing these concerns necessitates cultural sensitivity. This is as a fundamental aspect for being able to ask the right question, with a culturally appropriate content and in a culturally appropriate manner.^38^

### Answering several PROMs may be exhausting, annoying, or overwhelming

Psychosocial factors may make filling in PROMs hard. In our society the ability to read and write is taken for granted,^39^ and answering PROMs is expected to be unproblematic. One seems to forget that a small pile of questionnaires can seem overwhelming to any patient exhausted from chronic pain. Finding that a number of questions are more or less identical or do not reflect their particular experiences, may add frustration to the situation. On top of this, linguistic problems and/or poor reading skills may make a person feel helpless and overwhelmed. For some, also their psychosocial background may make it difficult to discuss certain topics.

## Conclusion

While the use of PROMs as tools for mapping and assessing symptoms and clinical care currently is growing, our findings demonstrate that cultural background and language skills may make filling in PROMs particularly challenging for migrant patients.

Although the PROMs used in this study were translated by professional translators, we found that cultural, religious and educational backgrounds influence how symptoms are expressed and how concepts and questions found in PROMs are understood, misunderstood, or perhaps not understood at all. Moreover, questions considered to be too private, or taboo might be ignored. Whatever the reason behind problems filling in PROMs, if not met, these problems may lead to seriously impaired treatment outcomes.

### Implications

A crucial factor of cultural sensitivity involves learning to pose the right question with a suitable content and in an appropriate manner.^38^ When healthcare personnel present migrant patients with a small stack of PROMs to work through, it is important that they allocate time to talk with them beforehand to explain why this task is important, even if it may be arduous, and that some questions might go against their sensibilities. Through this talk, they will gain some insight into how straightforward or difficult the task will be for the patient, and it will help patients ask questions and accept help from the healthcare personnel in order to cope with the task in hand.

After completing the PROMs, it is vital to clarify any misunderstandings. One should also inquire whether the patient has health problems that are not covered by the PROMs. During this talk, it is key to understand and validate the patients’ perspectives as well as explain the meaning of questions that is difficult for the patient to comprehend. This will boost mutual understanding and optimise the creation of person-centred treatment programmes.

### Strengths and limitations

We will hold that only users of the translations can truly validate their linguistic and cultural congruency. Hence, the inclusion of the four most common languages among the clinic’s migrant patient population was a purposeful sample strategy. That we were only able to enlist three interviewees from each language group, was somewhat disappointing. A strength of this study is the use of examples and quotations that illustrate challenges regarding responding to PROM questions.

Data saturation in qualitative research is debated. Ahmed^40^ holds that “[b]y including participants with varied backgrounds, experiences, or viewpoints, this approach ensures that saturation encompasses the breadth of the phenomenon under study.” Therefore, it is a strength that the participants come from diverse social, cultural and linguistic backgrounds. One obvious weakness is that only three patients from each language group were interviewed. A potential weakness is that the use of interpreters during four of the interviews may have influenced the conversations. According to Kapborga and Berterö,^41^ this may cause the quality of the data to be compromised, as nuances and meanings can be lost in translation.
